# An Ex Vivo Study on Release, Uptake, and miRNA Profile of Exosomes in Rat Lens

**DOI:** 10.1155/2022/6706172

**Published:** 2022-04-21

**Authors:** Junfang Zhang, Jiawei Luo, Guowei Zhang, Bai Qin, Xiumei Ren, Huaijin Guan

**Affiliations:** ^1^Eye Institute, Affiliated Hospital of Nantong University, Nantong, China; ^2^Shanghai Municipal Hospital of Traditional Chinese Medicine, Shanghai, China; ^3^Shanghai University of Traditional Chinese Medicine, Shanghai, China

## Abstract

**Purpose:**

To identify the ability of releasing and uptaking exosomes in rat lens and characterize the exosomal microRNA profile of lens-derived exosomes.

**Methods:**

The rat lenses were cultured ex vivo and the medium was collected. The exosomes were isolated from medium and measured in size and concentration by nanoflow cytometry (nFCM) and transmission electron microscopy (TEM) and verified with CD63 and TSG101 by Western blot. The miRNAs in exosomes released from lens epithelial cells (LECs) were sequenced. The plasma exosomes labeled by PKH26 were used to verify the exosomes uptake LECs, and their colocalized fluorescence was imaged by confocal microscopy.

**Results:**

LECs released numerous exosomes into the medium through the capsule, which contained abundant miRNAs. The most abundant miRNAs included miR-184, let-7c-5p, let-7a-5p, let-7b-5p, let-7f-5p, miR-125a-5p, miR-204-5p, miR-125b-5p, miR-1b, and miR-23a-3p. The LECs but not the lens fibre cells showed exosome uptake. The LECs uptake more PKH26-labeled exosomes at day 7 than day 3 and day 14.

**Conclusions:**

Our results suggested that LECs can release and uptake exosomes through the capsule. Exosomes may be an important way for the lens to communicate among LECs, aqueous humour, vitreous body, and other ocular tissues.

## 1. Introduction

The lens is an essential structure of the visual system whose primary function depends on its transparency and optical quality. Aging, UV-B exposure, and many other hazardous factors may cause lens opacity and clinically significant cataracts. Cataract is the leading cause of blindness globally. The pathogenesis of cataract remains unelucidated.

Exosomes are extracellular vesicles (EVs) containing various bioactive molecules, including protein, DNA, RNA, lipids, and sugars [1]. The vesicles can deliver biological signalling to nearby or remote recipient cells, which involved in a vast mechanism of cell-to-cell communication. It is well documented that exosomes may play a vital role in various ocular diseases [2, 3]. Previous studies reported that aqueous humour (AH) contained numerous exosomes with abundant miRNAs [4–6]. The exosomal miRNAs mediate in communication with AH inflow and outflow tissues [7]. Therefore, it is reasonable to hypothesize that the changes in AH miRNA levels may be associated with ocular function and disease.

Little is known about the origin of exosomes in AH. Theoretically, all cell types in contact with AH may contribute to the release or uptake of exosomes for cellular communication, including lens epithelial cells (LECs), ciliary epithelium, trabecular meshwork cells, corneal endothelial cells, and retinal neurons [5]. Wecker et al. also suggested that blood plasma contributes to the extracellular AH miRNAome [7]. Previous studies have shown that miR-184 and miR-204 increased simultaneously in LECs and AH exosomes of age-related cataract (ARC) patients [5]. The expression of miR-551b in AH exosomes is higher in diabetic cataracts than in ARC [6], indicating a possible involvement of exosomes in ARC pathogenesis.

The lens is enveloped by a thin membrane-like capsule which shapes it and prevents direct contact with other ocular tissues and the surrounding AH and vitreous humour (VH) [8]. Knowledge about the dynamics of exosomes across the capsule into AH is somewhat limited. To the best of our knowledge, no direct evidence showed that exosomes can be released from LECs to AH and VH freely through the capsule. In the present study, we used the rat lens ex vivo culture model to explore whether LECs can release and uptake exosomes through the capsule and aimed to provide direct evidence of exosome release and LECs uptake of exogenous exosomes. We further identified the miRNAs profile of the exosomes related to the lens in the ex vivo cultured condition. Since exosomes are of potential therapeutic value in eye disease and can be used as a biomarker for prognosis and diagnosis of ocular disease [9–12], our study may provide novel insights into their future application in the management of lens diseases.

## 2. Methods

### 2.1. Rat Lens Culture

The Animal Care and Use Committee of Nantong University approved the animal protocol of this study. The management of animal welfare followed the Association for Research in Vision and Ophthalmology statement for the use of animals in research. The rats lensed were cultured according to the previous studies [13, 14]. Adult Sprague-Dawley rats (220–240 g) were anesthetized with 30 mg/kg sodium pentobarbital intraperitoneal injection. Their eyes were enucleated, and the lenses were dissected carefully to avoid damage. Each lens was immediately transferred into 1 ml serum-free M199 medium (pH 7.2; Sigma, St. Louis, MO) with antibiotic mixture (Gibco BRL; 100 U/mL penicillin, 100 g/mL streptomycin, and 0.25 g/mL amphotericin B). Approximately 24 hours after lens preparation, transparent lenses were selected and cultured at 37°C in a 5% CO_2_ incubator for 14 days. The medium was collected daily and stored at −80°C for future use. Fresh medium was added into the culture daily.

### 2.2. Exosome Isolation

We used an exosome isolation kit (Umibio Co., Ltd., Shanghai, China) to enrich exosomes from the culture medium, following the manufacturer's instruction. After thawing at 25°C, the media were centrifuged at 3,000 g for 10 min at 4°C to remove cellular debris. The supernatants were collected and mixed with exosome concentration solution (ratio 4 : 1). The sample was homogenized using a vortex oscillator for 1 min and then incubated at 4°C for 2 h. The exosome pellets were obtained by centrifugation at 10,000 g at 4°C for 60 min and then resuspended in 100 *μ*l phosphate buffer solution (PBS). The exosomes isolated from the pooled medium of twenty lenses on the 3rd, 7th, and 14th day were used to measure size and concentration, and the exosomes isolated from the pooled medium of one hundred lenses for the 14 days were used for miRNA sequencing and Western blot.

### 2.3. Nanoflow Cytometry (nFCM) and Transmission Electron Microscopy (TEM)

The concentration, size, and distribution of exosomes were determined by nFCM according to the manufacturer's instructions (NanoFCM Inc., Xiamen, China) [15]. The isolated exosomes were diluted with distilled water at 1 : 2 ratio. The Silica Nanospheres Cocktail (S16M-Exo, NanoFCM Inc.) was employed as the size standard to calibrate size scattering intensity of vesicles.

The resuspended exosomes were fixed with 4% paraformaldehyde for 10 minutes. A 5 *μ*l volume of resuspended exosomes was transferred onto copper grids and dried for 5 minutes at room temperature. One drop of 2% uranyl acetate solution was then added for the fixation for 1 minute, followed by TEM observation (JEM-1200EX, JEOL Ltd, Japan).

### 2.4. Protein Preparation and Western Blot Assays

The capsules (containing LECs) were dissected from rat lenses under the microscope. RIPA buffer (Beyotime, Shanghai, China) was used in isolation of protein from exosomes and LECs. The LECs proteins were used as positive control. The loading amount of each lane was 10 *μ*g. Antibodies against TSG101 (1 : 1500, Abcam, Cambridge, UK) and CD63 (1 : 1500, Abcam) were used as primary antibodies. The horseradish peroxidase-linked anti-rabbit IgG (1 : 5000, Abcam) was used as secondary antibodies. Detail operation of Western blot was described in our previous studies [16].

### 2.5. Exosomal RNA Extraction and miRNA Sequencing

Total RNA was extracted from exosome pellets using the TRIzol reagent (Life Technologies, Carlsbad, CA, USA). Small RNA libraries were prepared and amplified using the TruSeq™ Small RNA sample prep kit (Illumina Inc., San Diego, CA) according to the manufacturer's protocol. Small RNA was sequenced at SR50 Rapid Run Mode on Illumina Hiseq2500. The sequences of mature miRNA and precursor miRNA for *Rattus norvegicus* from miRBase version 21 (http://www.mirbase.org/) were used to identify miRNA sequences.

### 2.6. Plasma Exosome Preparation and Fluorescence Labelling

Plasma exosomes were isolated to test exosome uptake of lens, with 10 ml of citrated blood from each rat used for exosome preparation. The whole blood was centrifuged at 1,600 g for 15 min at 4°C to obtain plasma. Two-steps of enrichment were adopted for further differentiation and centrifugation at 10,000 g for 30 min and the supernatant were centrifugated again at 100,000 g for 70 min at 4°C. The concentrated exosomes were labeled using the red lipophilic fluorescent dye, PKH26 (PKH26GL, sigma, USA) for 5 min at room temperature with 1% bovine serum albumin (BSA) to stop the staining reaction. PKH26-labeled exosomes were pelleted at 100,000 g for 70 min, washed three times with PBS, and resuspended in serum-free M199 medium. The lenses were incubated with PKH26-labeled exosomes for 48 hours at different times using PBS as the blank control. The frozen lens sections were made following a previously reported procedure [17]. The nuclei were stained with Hoechst dye for 5 minutes. The distribution of exosome within the lenses was observed using confocal microscopy (Leica TCS SP5, Germany).

## 3. Results

### 3.1. Characteristic of Exosomes Released from Rat Lens

**Figure 1:**
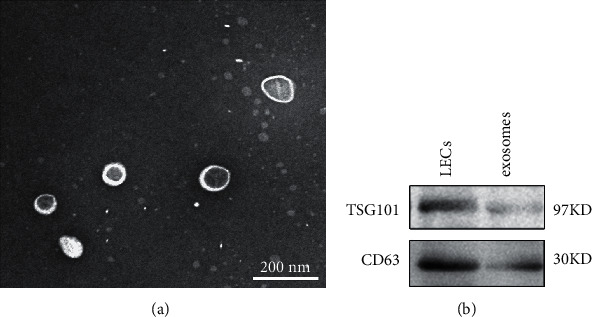
Characteristic of exosomes released from lenses. (a) Transmission electron micrograph of exosomes isolated from ex vivo rat lenses culture media at day 7. Scale bar = 200 nm. (b) CD63 and TSG101 expressed in LECs and exosomes from rat lenses were detected by Western blot.

**Figure 2:**
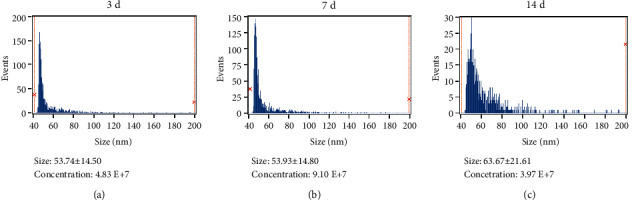
Concentration and size of the exosomes isolated from culture medium of rat lenses were measured using nFCM. (a) Exosomes isolated from the pooled medium of ten rat lenses at day 3. (b) Exosomes isolated from the pooled medium of ten rat lenses at day 7. (c) Exosomes isolated from the pooled medium of ten rat lenses at day 14.

The exosomes were isolated from the medium of rat lenses cultured ex vivo. TEM clearly revealed that the exosomes had a typical cup-shaped morphology and a characteristic central depression (Figure 1(a)) with a diameter of about 50–60 nm. Western blot showed that CD63 and TSG101 (markers of exosomes) expressed in the LECs and exosomes of rat lenses (Figure 1(b)). The exosome concentrations were 4.83 × 10^7^, 9.10 × 10^7^, and 3.97 × 10^7^ particles/ml at day 3, 7, and 14, respectively. The peak diameters were 53.74 ± 14.50 nm, 53.93 ± 14.80 nm, and 63.67 ± 21.61 nm, respectively (Figures 2(a)–2(c)).

### 3.2. The Profile of Exosomal miRNA Released from LECs

**Figure 3:**
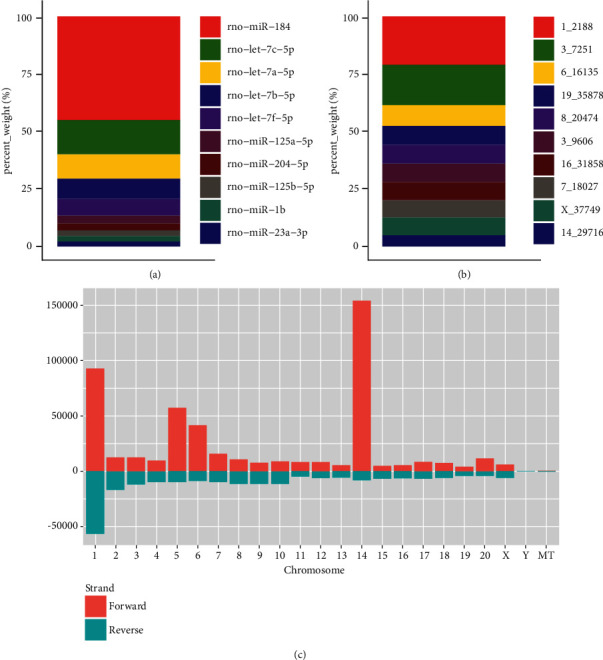
miRNA profile of exosomes isolated from ex vivo rat lens culture medium. (a) The top ten known miRNAs in the exosomes released by LECs. (b) The top ten novel miRNAs in the exosomes released by LECs. (c) Chromosome distribution of exosomal miRNAs.

miRNA sequencing was used to profile the miRNAs distribution in exosomes isolated from the pooled medium. We identified 145 miRNAs in exosomes with the ten most abundant miRNAs being miR-184, let-7c-5p, let-7a-5p, let-7b-5p, let-7f-5p, miR-125a-5p, miR-204-5p, miR-125b-5p, miR-1b, and miR-23a-3p (Figure 3(a)). Of 384 novel miRNAs detected in exosomes, the ten most abundant were 1_2188, 3_7251, 6_16135, 19_35878, 8_20474, 3_9606, 16_31858, 7_18027, X_37749, and 14_29716 (Figure 3(b)). Chromosome analysis revealed that the 145 known miRNAs were located within almost all chromosomes except for the mitochondrial chromosome. Chromosome 14 contained a relatively large number of exosomal miRNAs of LECs (Figure 3(c)).

### 3.3. Ingestion of Exogenous Exosomes in LECs

**Figure 4:**
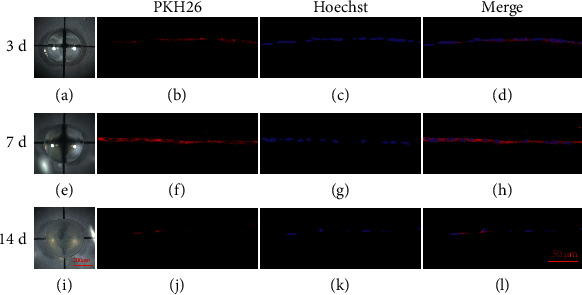
Uptake of PKH26-labeled exosomes by LECs sub anterior capsule of lenses cultured ex vivo. (a, e, i) Photographs of lenses cultured ex vivo for 3 days, 7days and 14 days. Scale bar 1 = 200 μm. (b–d) Fluorescent photographs of frozen sections from lenses cultured for 3 days. (f–h) Fluorescent photographs of frozen sections from lenses cultured for 7 days. (j–l) Fluorescent photographs of frozen sections from lenses cultured for 14 days. Red represents PKH26; blue represents the nucleus. Scale bar 2 = 50 μm

**Figure 5:**
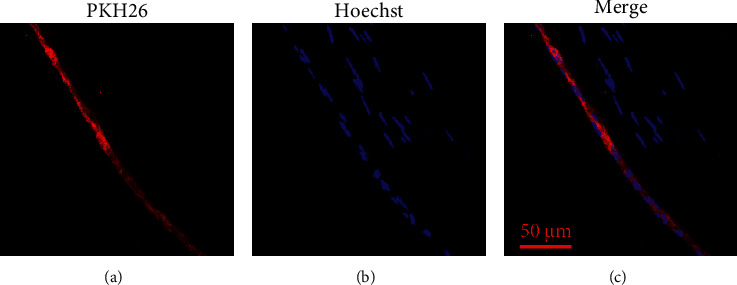
Equatorial LECs uptake of PKH26-labeled exosomes was detected using confocal microscopy after lens cultured ex vivo for 7 days. Red represents PKH26; blue represents the nucleus. Scale bar = 50 μm

During the lens culture process, the lenses were transparent at day 3, mildly turbid at day 7, and opaque at day 14 (Figures 4(a), 4(e), 4(i)). Lens uptake of PKH26-labeled exosomes was higher at day 7 than day 3 and day 14 (Figures 4(b), 4(f), 4(j)). The number of LECs in the anterior capsule decreased at day 14. Exosomes uptake decreased during the culture duration (Figures 4(c), 4(j), 4(k)). The equatorial LECs but not nucleated lens fibre cells engulfed the PKH26-labeled exosomes (Figures 5(a)–5(c)).

## 4. Discussion

Using rat lenses cultured ex vivo, this study provides direct evidence that LECs can release and engulf exosomes through the lens capsule. The size and concentration of the exosomes released from LECs were measured using nFCM. Studies have shown that NTA has a detection limit of 70–90 nm [18] and overestimates EVs size by about 50–100 nm compared to TEM [19]. We used nFCM because it is a laboratory-built method with minimum detectable EV size of 40 nm [15]. We found that the peak size of exosomes released from LECs is between 50 and 65 nm, and these findings were supported by TEM imaging results, but are smaller than estimates from previous studies. We cannot make a direct comparison and the different methodologies may explain this discrepancy. We speculated that the changes in exosomal size and concentration on the 14th day may be associated with the LEC loss and lens enlargement after lens opacity.

Previous studies have discussed a change in AH exosome content in cataract patients but did not exactly identify the origin of the exosomes [6]. While almost all cells in contact with the AH may release exosomes into it, there are insufficient data to support a single responsible tissue/cell type [5]. The sizes of exosomes released from LECs are smaller than those of exosomes from other cells including those of the AH. The selective passage of intermediate-sized molecules through the lens capsule is dependent on their size and charge [20]. We speculate that only exosomes with diameters smaller than the aperture of the capsule can breach the lens freely. This idea remains to be tested in the future.

Our study investigated the exosomal miRNA profile based on small RNA sequencing. The exosomes contained abundant miRNAs, including 145 known miRNAs and 384 novel miRNAs. miR-184, let-7 family, miR-204, and miR-125a and b were prevalent in the exosomes isolated from the rat lens culture media. These prevalent miRNAs in exosomes of rat lens were strongly expressed in LECs, AH, or AH exosomes [7, 21, 22]. Among them, miR-204 and let-7b were positively correlated with lens opacity [23, 24]. We summarised some of the previously published findings on miRNA profile in LECs, AH, and AH exosome samples [5, 7, 21, 22], as given in Table 1. Our data strongly suggest that exosomal miRNAs released from LECs contain an important set of AH “background miRNAs” of LECs. Exosomal miRNAs released from LECs may be involved in information exchange between the lens and AH.

Intercellular exosome exchanges have been previously reported [25]. The ingestion of exosomes by recipient cells may follow a pattern of nonselective incorporation from donor cells into recipient cells, but the exosome uptake mechanisms vary depending on the recipient cell types [26], including direct fusion, receptor-mediated interactions, and endocytosis [25]. Some studies have demonstrated the uptake of exosomes from specific ocular cells. Exosomes secreted by human corneal epithelial cells may be taken up by corneal fibroblasts, while the epithelial cells may engulf exosomes released from human mesenchymal stromal cells [12, 27]. These studies provided a theoretical basis for the clinical application of exosomes in ocular diseases. LECs are wrapped in the lens capsule and not directly in contact with aqueous humour. Our study provides the first evidence that ex vivo LECs can take up exosomes from their medium environment through the capsule, but not the lens fibre cells. However, questions regarding whether the capsule size and charge limit exosome passage and how LECs take up the exosomes remain to be addressed by future research.

In summary, we identified for the first time that the lens can release and take up exosomes through its capsule and identified the miRNA profile of exosomes released from rat lens cultured ex vivo. miR-184 and Let-7 family are highly abundant in exosomes derived from LECs. Our study suggests that exosomes may be an important player in communication among the lens, AH, and other ocular cells.

## Figures and Tables

**Table 1:** The top miRNAs expression in human LECs, AH, and AH exosomes.

Study	Sample	Method	miRNA amount	Top abundant miRNAs
Wu et al., 2012	Human LECs	miRNA array	206	miR-184, miR-1826, let-7b/c, miR-24, miR-23b, miR-923, and miR-23a
Dunmire et al., 2013	Human AH	miRNA array	110	miR-202, miR-193b, miR-135a, miR-365, miR-376a, miR-486-5p, miR-188-5p, miR-195, miR-431, and miR-16
Wecker et al., 2016	Human AH	miRNA sequence	158	miR-451, miR-184, miR-4448, miR-21, miR-26, miR-16, miR-19, miR-101, miR-205, and miR-3074
Dismuke et al., 2015	Human AH exosome	miRNA sequence	>10	miR-486-5p, miR-184, miR-204, miR-181a, miR-191, miR-148a, miR-26a, miR-125a-5p, let-7a, and let-7b.

## Data Availability

The data used to support the findings of this study are included within the article.
